# ACE phenotyping in human heart

**DOI:** 10.1371/journal.pone.0181976

**Published:** 2017-08-03

**Authors:** Victoria E. Tikhomirova, Olga A. Kost, Olga V. Kryukova, Elena Z. Golukhova, Naida I. Bulaeva, Aigerim Z. Zholbaeva, Leo A. Bokeria, Joe G. N. Garcia, Sergei M. Danilov

**Affiliations:** 1 Chemical Faculty, M.V. Lomonosov Moscow State University, Moscow, Russia; 2 Bakulev Center for Cardiovascular Surgery, Moscow, Russia; 3 University of Arizona Health Sciences, Tucson, Arizona, United States of America; 4 Department of Anesthesiology, University of Illinois at Chicago, Chicago, Illinois, United States of America; Max Delbruck Centrum fur Molekulare Medizin Berlin Buch, GERMANY

## Abstract

**Aims:**

Angiotensin-converting enzyme (ACE), which metabolizes many peptides and plays a key role in blood pressure regulation and vascular remodeling, is expressed as a type-1 membrane glycoprotein on the surface of different cells, including endothelial cells of the heart. We hypothesized that the local conformation and, therefore, the properties of heart ACE could differ from lung ACE due to different microenvironment in these organs.

**Methods and results:**

We performed ACE phenotyping (ACE levels, conformation and kinetic characteristics) in the human heart and compared it with that in the lung. ACE activity in heart tissues was 10–15 lower than that in lung. Various ACE effectors, LMW endogenous ACE inhibitors and HMW ACE-binding partners, were shown to be present in both heart and lung tissues. “Conformational fingerprint” of heart ACE (i.e., the pattern of 17 mAbs binding to different epitopes on the ACE surface) significantly differed from that of lung ACE, which reflects differences in the local conformations of these ACEs, likely controlled by different ACE glycosylation in these organs. Substrate specificity and pH-optima of the heart and lung ACEs also differed. Moreover, even within heart the apparent ACE activities, the local ACE conformations, and the content of ACE inhibitors differ in atria and ventricles.

**Conclusions:**

Significant differences in the local conformations and kinetic properties of heart and lung ACEs demonstrate tissue specificity of ACE and provide a structural base for the development of mAbs able to distinguish heart and lung ACEs as a potential blood test for predicting atrial fibrillation risk.

## Introduction

Atrial fibrillation (AF) is the most common cardiac arrhythmia, causing substantial cardiovascular morbidity and mortality [1]. Although risk factors have been described, there are no available blood tests to predict AF risk.

The activation of renin-angiotensin system (RAS) definitively plays an important role in the pathogenesis of atrial fibrillation [2–4]. The angiotensin II is a primary mediator of RAS which is mainly produced from angiotensin I by angiotensin-converting enzyme (ACE, EC 3.4.15.1, CD143). Patients with AF are known to have increased level of ACE expression in the heart tissue, in particular, in atria [2]. Study on the transgenic mice also demonstrated that the increased ACE expression in the heart might be a causative factor for AF and sudden cardiac death [5]. Both ACE inhibitors and angiotensin receptor blockers reduce AF incidence and may prevent AF-related complications in patients and in experimental models [3].

ACE is a Zn^2+^ peptidyldipeptidase which plays key roles in the regulation of blood pressure by producing angiotensin II and degrading bradykinin and in the development of vascular, including cardiac, pathology and remodeling. ACE is constitutively expressed on the surface of endothelial and some epithelial cells, as well as cells of the immune system (macrophages, dendritic cells) reviewed in [6,7]. ACE expression in the normal and pathological human hearts has been studied previously [8,9]. To date, several new substrates for ACE have been identified (AcSDKP, angiotensin 1–7) and new functions for ACE have been proposed, such as outside-in cell signaling, antigen presentation [10–12]. The anti-fibrotic and anti-inflammatory actions of a substrate for ACE, AcSDKP, seem to be especially important for AF pathogenesis in patients with the enhanced ACE level in the heart [13].

Despite the strong relationship of RAS activation to arrhythmias, plasma levels of ACE do not correlate to AF and ventricular arrhythmias. Generally, plasma ACE accurately reflects the level of tissue ACE [14]. Blood ACE originates from endothelial cells, mostly lung capillaries, which exhibit nearly 100% ACE expression compared to only 5–15% ACE-positive capillaries in the systemic circulation [15]. Based on that, we estimated that ACE shed from heart capillaries could represent not more than 1% of total ACE in the blood. This is why a total plasma ACE level in patients does not seem to be a predictive parameter for patients with AF. However, as atrial ACE increases 3-fold in patients with AF [2], the quantification of the definite heart-derived ACE (mainly, atria-derived ACE) in plasma theoretically could be used as a predictive test for the risk of AF.

We proved the concept that the conformation of ACE is cell- and tissue-specific and stems likely from different glycosylation of the enzyme on the example of ACE from lung endothelial cells versus ACE from macrophages and dendritic cell of sarcoid granuloma [16] and ACE from epithelial cells of prostate [17]. The ACE conformational fingerprint based on the pattern of the binding of a set of mAbs to different epitopes on the surface of ACE [16] has, therefore, a potential for the disclosure of the cells/organs from which ACE originates.

We applied this approach here to demonstrate conformational differences of ACEs originated from human heart and lung and showed differences for purified ACEs, for ACEs in tissue homogenates, and ACEs in the blood after *in vivo* perfusion. We believe that such differences will allow the generation of monoclonal antibodies (mAbs) able to distinguish ACEs shed to the blood circulation from these two organs and, therefore, form the base for the blood test for predicting the risk of AF.

## Experimental section

### ACEs from different sources

The work was carried out in accordance with The Code of Ethics of World Medical Association (Declaration of Helsinki) and was approved by the Institutional Review Boards of the Bakulev Center of Cardiovascular Surgery, Moscow State University, and the University of Illinois at Chicago. None of the donors were from the vulnerable populations and all donors or next of kin provided written informed consent that was freely given. Human citrated plasma and tissue homogenates (1:9 and, in some cases, 1:3 w/v ratio) from 10 donors (five male donors, age 54–66, and five female donors, age 28–68), including three donors with atrial fibrillation, were used as sources of somatic ACEs. Lung and heart ACEs were purified from tissue homogenates using anion-exchange chromatography on DEAE-Toyopearl 650M and then lisinopril affinity chromatography—as in [18] “S1 Table”. Purified ACE preparations were proved to be homogeneous by electrophoresis in 7.5% SDS-PAGE “S1 Fig”.

### ACE activity assay

ACE activity in blood plasma, homogenates of human organs or homogenates of heart chambers was measured using a fluorimetric assay with two ACE substrates, 2 mM Z-Phe-His-Leu (ZPHL) and 5 mM Hip-His-Leu (HHL) [19]. Inhibition of ACE activity with anti-catalytic mAb 5F1 to the N domain of ACE and mAb 1E10 to the C domain was performed at mAbs concentrations 100 μg/μl and 10 μg/μl, correspondingly, with 1mM ZPHL or 2.5 mM HHL as substrates.

### Substrate specificity of ACE

The kinetic parameters of the hydrolysis of several synthetic tripeptide substrates and natural substrate decapeptide angiotensin I by purified human heart and lung ACEs were determined in 0.05 M phosphate buffer, pH 7.5, containing 0.15 M NaCl and 1 μM ZnCl_2_, at 25°C. The rates of enzymatic hydrolysis of Z-Phe-His-Leu, Hip-His-Leu and angiotensin I were determined fluorimetrically, whereas the kinetics of the hydrolysis of FA-containing substrates, FA-Phe-Gly-Gly and FA-Phe-Phe-Arg, was studied spectrophotometrically [20].

### Immunological characterization of ACE (Plate immunoprecipitation assay)

Ninety six-well plates (Corning, Corning, NY) were coated with anti-ACE mAbs via goat anti-mouse IgG (Pierce, Rockford, IL) bridge [21] and incubated with different sources of ACE, which were equilibrated for ACE activity. After washing off unbound ACE, plate-bound ACE activity was measured by adding a substrate for ACE, Z-Phe-His-Leu, directly into the wells [21]. Sixteen mAbs to human ACE were generated in our lab [16], while mAb BB9 was kindly provided by Paul J. Simmons (then Brown Foundation of Molecular Medicine, University of Texas Health Science Center, Houston, TX, USA).

### Dialysis and filtration of human plasma and heart and lung homogenates

Dialysis of the heart and lung homogenates was performed in 10 kDa dialysis cassettes (Pierce, Rockford, IL) and in 100 kDa Biotech dialysis tubes (Spectrum Inc., Houston, TX) against 0.05 phosphate buffer, pH 7.5, 0.15 M NaCl and 1 μM ZnCl_2_, at 4°C. Filtration of the homogenates was performed on Vivaspin filtration membranes (GE Healthcare, Sartorius Corp., Bohemia, NY) with 30 kDa and 100 kDa limits at 12 000g.

### Aminopeptidase activity

Aminopeptidase activity in tissue homogenates at different dilutions was estimated by the rates of the hydrolysis of 0.01–0.1 mM His-Leu in PBS-BSA buffer, pH 8.3, at 37°C.

### ACE perfusion in rats

All *in vivo* rat methods/experiments were approved by and performed in accordance with Moscow State University Committee on the Ethics of Animal Experiments guidelines and regulations that conform to the NIH guidelines (Guide for the care and use of laboratory animals). Adult male Wistar rats weighing ~300 g were put under pathogen-free condition in individual plastic cages with sawdust bedding in an air-conditioned room under constant temperature (23±1°C) and 70% humidity. Rats were provided with water and standard diet (LabDiet, 5053 (LabDiet; St. Louis, MO, USA)) *ad libitum*. All animals were monitored for body health on a daily basis for a week before an experiment. All efforts were made to minimize rats suffering. Purified human heart and lung ACEs, 1000 mU in 100 μl, pH 7.4, were injected into the tail vein of rats (two rats per group) with anesthesia with ketoprofen. After 30 min, euthanasia was performed by decapitation, the blood was collected and citrate plasma prepared. At that moment, about 30% of human ACE was still in the rat blood. Precipitation of human ACE from rat plasma by a set of mAbs to ACE was performed as described above but corrected for trace precipitation of the rat ACE by these mAbs.

### Statistical analysis

All data are means ± SEM. Significance was analyzed using the Mann-Whitney test with STATISTICA 6 (StatSoft, Inc., OK).

## Results and discussion

### ACE activity in the human heart

**Fig 1:**
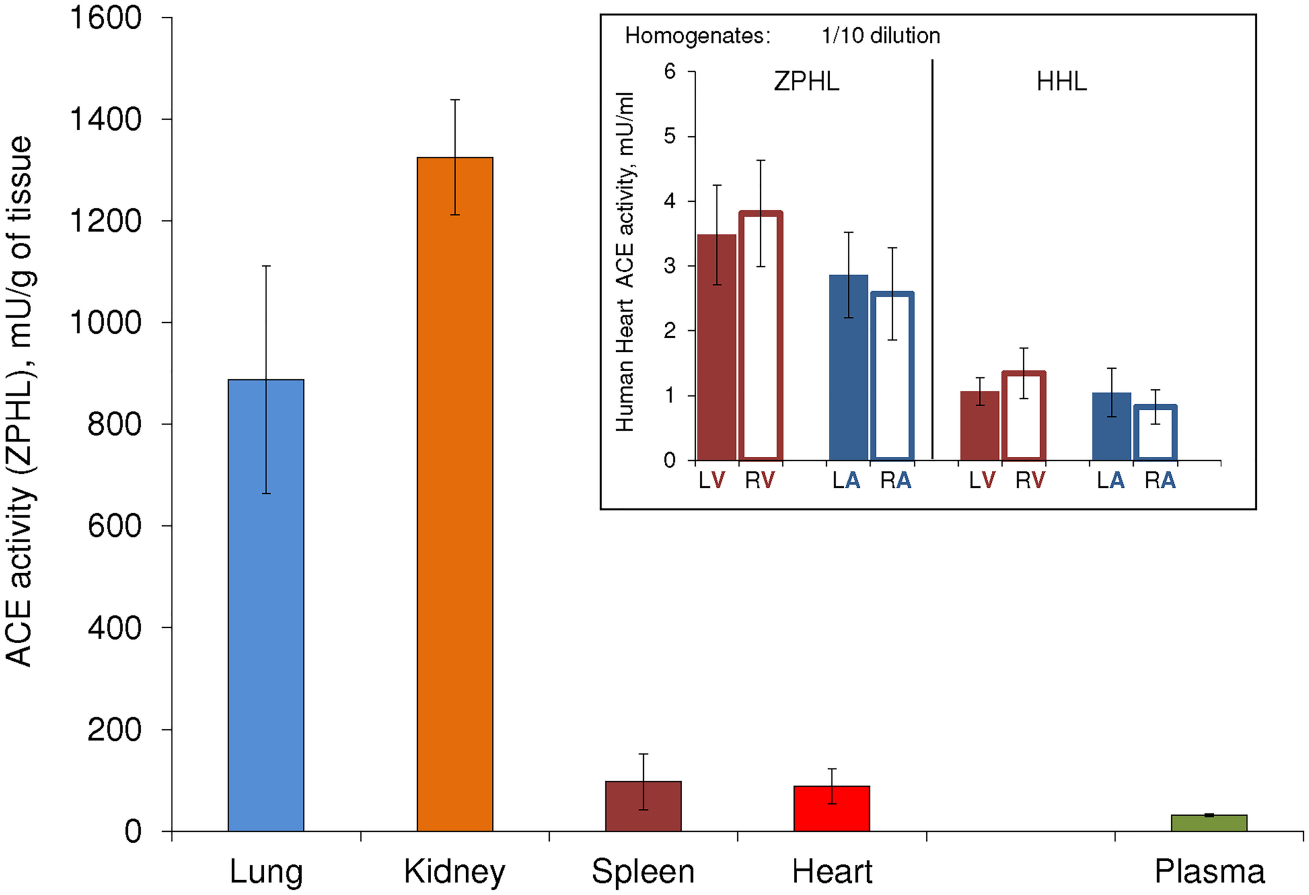
ACE activity in human tissues. ACE activity in tissue homogenates from 10 donors and citrated plasma was quantified using a spectrofluorimetric assay with 2 mM ZPHL and 5 mM HHL as substrates. Homogenates were prepared at 1:9 ratio (weight/volume) and further diluted 1/10 to minimize an effect of putative endogenous ACE inhibitors and LMW ACE effectors. Plasma was diluted 1:4 with PBS. Data are expressed as mU per gram of tissue (for homogenates) or per ml of undiluted plasma, p<0.01. Insert: ACE activity (mU/ml) in homogenates of different chambers of human hearts from 10 donors. V-ventricles; A-atria; L-left; R-right. Each value is a mean of several (2–3) experiments in duplicates, p<0.05.

We estimated ACE expression in different human tissues, namely, heart, lung, kidney and spleen, by the comparison of ACE activity in homogenates of these tissues as well as in human citrated plasma. ACE activity in the human heart homogenate (expressed in mU per gram of tissue) was about 3-fold more than in human plasma and 8-12-fold less than in human lung (Fig 1). This estimation correlates with the density of radioligand ACE inhibitor binding sites in human heart and lung [22], as well as with high ACE RNA transcription in the lung, while negligible in the heart, as in Human Protein Atlas [23].

ACE activity measured simultaneously by two substrates in the homogenates of different whole heart chambers showed statistically significant difference in atrial and ventricular ACE activity, this activity being the highest in the right ventricle and the lowest in the right atrium (Fig 1, insert).

**Fig 2:**
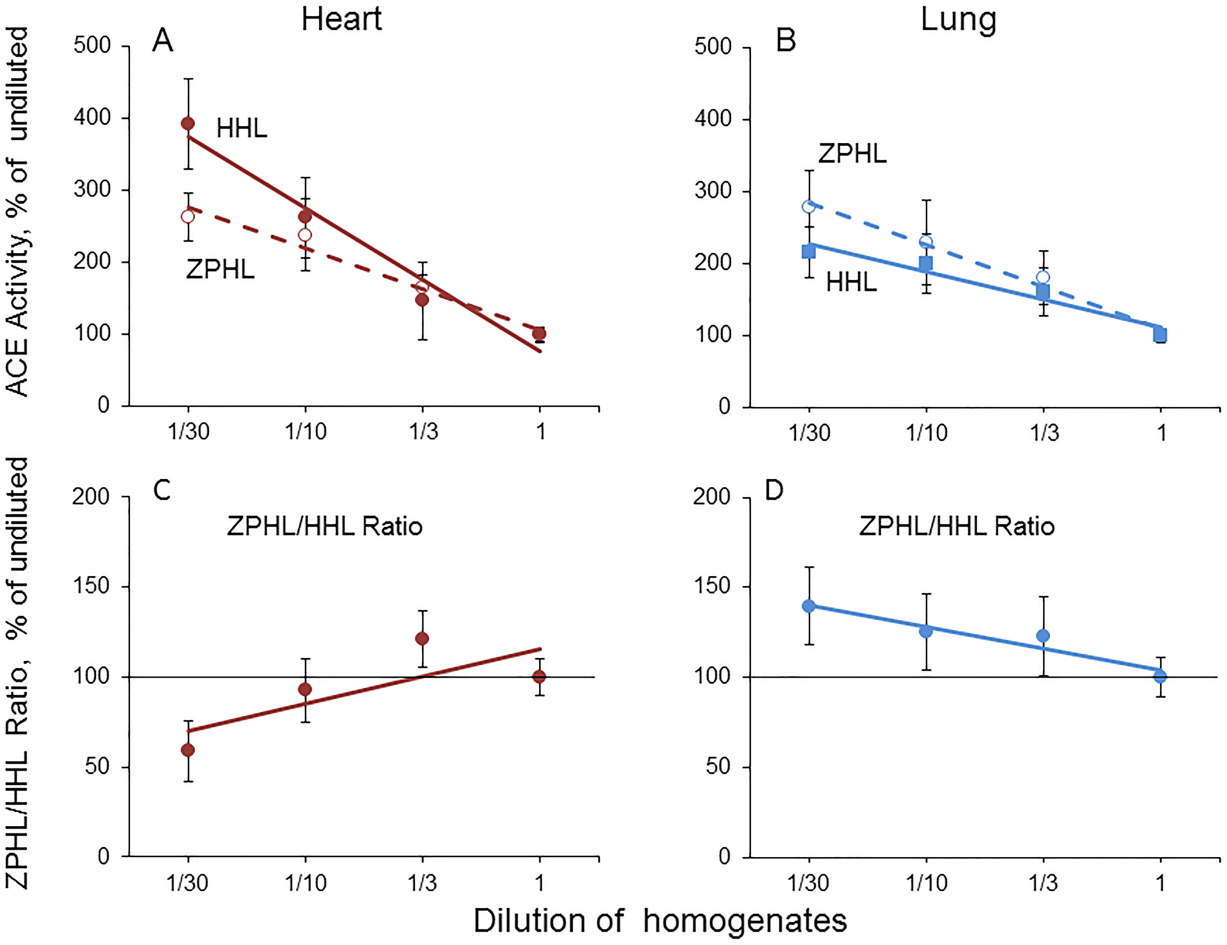
Effect of dilution on the apparent ACE activity in the heart and lung homogenates. ACE activity was measured in the heart and lung homogenates from 10 donors at different dilutions using two substrates, ZPHL and HHL (as in the legend to Fig 1). Data are expressed as % from the ACE activity in undiluted homogenates (**A,B**), as well as % of ZPHL/HHL ratio from that for undiluted homogenates (**C**,**D**). Each value is a mean of several (2–3) experiments in duplicates, p<0.01.

Mammalian tissues and blood contain endogenous ACE inhibitors [17,24,25] and ACE effectors [17,26], including putative ACE binding proteins [26–28]. In order to demonstrate the presence of endogenous ACE inhibitors in human tissues we compared an apparent ACE activity in the heart and lung homogenates at serial dilutions (Fig 2). An apparent ACE activity significantly increased 3-4-fold during dilution of both homogenates (Fig 2A and 2B). We excluded the presence of aminopeptidases in human tissues as a reason for that effect “S1 Text”. Thus, an effect of dilution reflects the presence of endogenous ACE inhibitors in heart and lung tissues. Dialysis (10 or 100 kDa) and filtration (100 kDa) of the heart and lung homogenates also resulted in a similar (200–400%, p<0.05) increase of an apparent ACE activity (data not shown). Therefore, in order to estimate correctly ACE activity in the heart (or lung) homogenate it is not necessary to perform time-consuming dialysis, as simple dilution of homogenates 10-fold successfully induces dissociation of low molecular weight (LMW) ACE endogenous inhibitors from their complex with ACE.

We discovered that the effect of the dilution of tissues homogenates on relative ACE activity with two substrates, Z-Phe-His-Leu and Hip-His-Leu (ZPHL/HHL ratio), was substrate specific (Fig 2A and 2B). We have shown previously that the selective inactivation/inhibition of the C domain increases ZPHL/HHL ratio for somatic, two-domain ACE, whereas selective inactivation/inhibition of the N domain decreases this ratio, as these substrates are cleaved by the two domains with different rates [19]. Therefore, we can interpret the decrease of ZPHL/HHL ratio (Fig 2C) and increase of this ratio (Fig 2D) upon dilution of heart and lung homogenates, respectively, as an evidence of the presence of different sets of endogenous ACE inhibitors in the heart and lung tissues.

### Conformational fingerprinting of heart and lung ACEs

**Fig 3:**
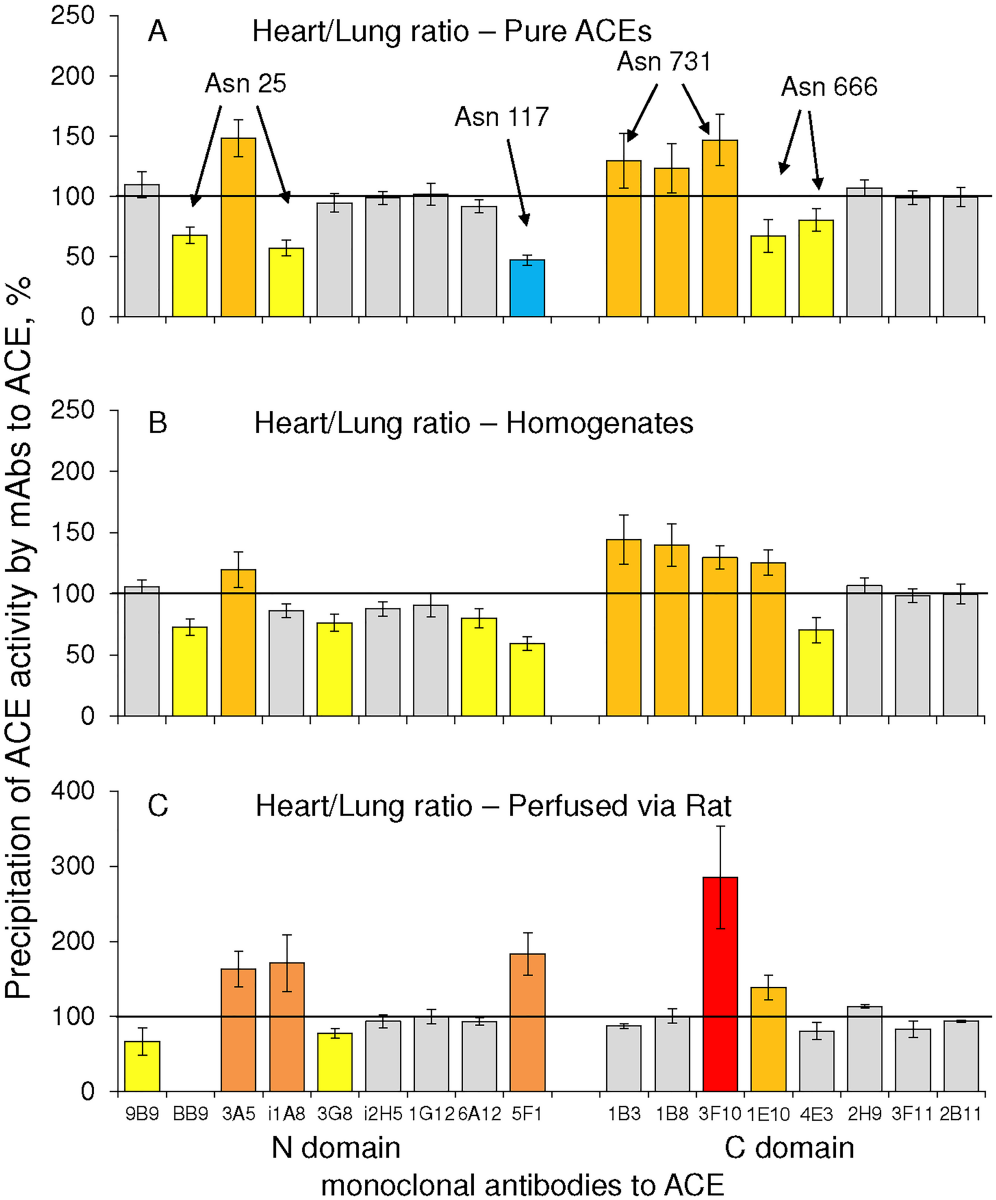
Conformational characteristics of different ACEs. Conformational fingerprinting of the heart and lung ACEs was performed with a set of 17 mAbs to the two-domain ACE. Immunoprecipitated ACE activity from purified ACEs solutions (**A**), tissue homogenates from 10 donors (**B**), or ACEs after perfusion into rat blood circulation (**C**) are presented as % (“binding ratio”) for heart ACE from that of lung ACE. Ratios increased more than 20% are highlighted in orange, more than 50% in dark orange, and more than 200% in red, while decreased more than 20% are highlighted in yellow and more than 50% in deep blue. Data are mean ± SD of at least 3 experiments (each in duplicates), p<0.01.

We characterized the conformation of the heart and lung ACEs using a panel of mAbs directed against 17 different epitopes and mapped on the surface of the N and C domains of catalytically active human ACE–a method of “conformational fingerprint of ACE” [16]. As apparent in Fig 3, the immunoprecipitation profile of heart ACE significantly differed from that for lung ACE, the difference being observed both for the purified enzymes (Fig 3A) and ACEs in homogenates (Fig 3B). This allowed us to conclude that heart ACE, originated from heart endothelial cells and perhaps myofibroblasts [9] and lung ACE, originated from lung endothelial cells [15,29], exhibit different local conformations of their surface, probably, due to tissue- and cell-specific post-translational modifications (PTM). PTM, which are common for various proteins, can precisely regulate the functions of proteins by inducing conformational changes, which subtly or dramatically alter the protein surface or its overall tertiary structure. The most common PTM is glycosylation [30].

Somatic ACE represents a type-I N-glycosylated membrane glycoprotein, exact glycan structures of which, as well as locations of actually present oligosaccharide chains on the surface of the protein globule, can vary with a protein source. The amino acid sequence of human somatic ACE contains 17 potential sites for N-glycosylation [31]. The structure and exact positions of glycan moiety in human somatic ACE from different tissues were not investigated completely [17,32].

Heart and lung ACEs possess rather equal masses about 170 kDa as was revealed by SDS-PAGE “S1 Fig”which indicates on the absence of major differences in the degree of glycosylation of these ACEs. It was shown earlier that common sialylated biantennary complex oligosaccharide can contact the surface of the enzyme within an area about 200–300 Å [17]. As epitopes for mAbs are usually 600–900 Å, it is obvious that the presence of the oligosaccharide within an epitope (as well as its definite structure, i.e. the number of antennae, sialylation, fucosylation, etc.) can hugely influence mAbs binding. Previously, we showed that different glycosylation of ACE in endothelial and epithelial cells could be the main reason for the differences in mAbs binding to ACEs from lung and seminal fluid [17]. Moreover, the difference in binding efficiency for some particular mAb can be attributed to the different glycosylation of the corresponding glycosylation site within epitope for this very mAb. So, the main reason for the difference observed for immunoprecipitation profiles of the heart and lung ACEs (Fig 3) could be due to different glycosylation of ACE, even in endothelial cells but from different organs, induced by different environment of these cells in different organs. Conformational fingerprints of heart and lung ACEs (Fig 3A and 3B) allowed us to suggest that different glycosylation of these ACEs occurs in the following glycosylation sites on ACE: Asn25 in the epitopes for mAbs BB9, 3A5 and i1A8, and Asn117 in the epitope for mAb 5F1 on the N domain, as well as Asn666 in the epitope for mAbs 4E3 and 1E10, and Asn731, which is within the epitopes for mAbs 1B8 and 3F10 and close to the epitope for mAb 1B3 on the C domain of ACE.

The purified heart and lung ACEs were isolated from different donors and strictly speaking the differences in mAbs binding to these purified ACEs (Fig 3A) could be attributed to the inter-individual differences in the protein glycosylation in these donors [33], in our case, ACE glycosylation. In order to exclude this possibility, we performed the conformational fingerprints of heart and lung ACEs in homogenates obtained from the same donor. For statistical reasons and reproducibility, we used 10 pairs of homogenates obtained from the tissues from 10 donors. As the general pattern of mAbs binding to ACE in pairs of homogenates (Fig 3B) was similar to the pattern of mAbs binding to purified heart and lung ACEs from different donors we can conclude that different conformational fingerprints of heart and lung ACEs demonstrate a real conformational tissue specificity of the enzyme. It is worth noting that ACE from donors with AF in anamnesis demonstrated similar differences in mAbs binding to heart and lung ACEs (not shown), that is this difference is not caused by the disease but by the nature of ACE-producing cells.

In order to confirm that the differences in local ACE conformation of heart and lung ACEs retain in the blood we perfused purified ACEs from heart and lung (and seminal fluid ACE as a representative of ACE produced by epithelial cells) via alive rat circulation. Circulation of glycoproteins (including ACE) in blood after shedding from the cell membranes leads to the enrichment of serum glycoproteins with molecules with a higher content of sialic acid residues as a result of the selective removal of asialo-molecules (with terminal galactosyl residues) by hepatic lectin, while sialylated glycoproteins remain in the circulation [34]. The results clearly demonstrate that the effect of such perfusion on the pattern of mAbs binding to heart and lung ACEs is significantly different, once again demonstrating that the patterns of glycosylation (sialylation, in particular) of the heart and lung ACEs are different. In addition, the binding of mAbs 5F1 and 1E10, which better bind to ACE molecules when glycan in the epitopes for these mAbs is less sialylated (Danilov and Trakht, unpublished observation), decreased after perfusion of the lung ACE into the rat in accordance with expected relative enrichment of ACE moiety with sialic acids. As a result of the perfusion of the heart ACE, however, the binding of these very mAbs unexpectedly increased, which indirectly confirms that the local conformation of heart ACE and its sensitivity to the structure of glycan differs from that for lung ACE.

It is important, that even after circulation in the blood, the immunoprecipitation profiles of soluble ACEs from heart and lung remained significantly different (Fig 3C and “S2 Fig“) proving the possibility to distinguish ACEs produced by different organs in a real blood.

Therefore, an analysis of ACE conformation provides a structural base for the generation of mAbs able to distinguish ACE produced by a definite cell type or definite organ, in particular, by heart, and to generate a blood test for the detection and quantification of this particular ACE in the blood for the prediction of AF risk.

### ACE effectors in human heart tissue

**Fig 4:**
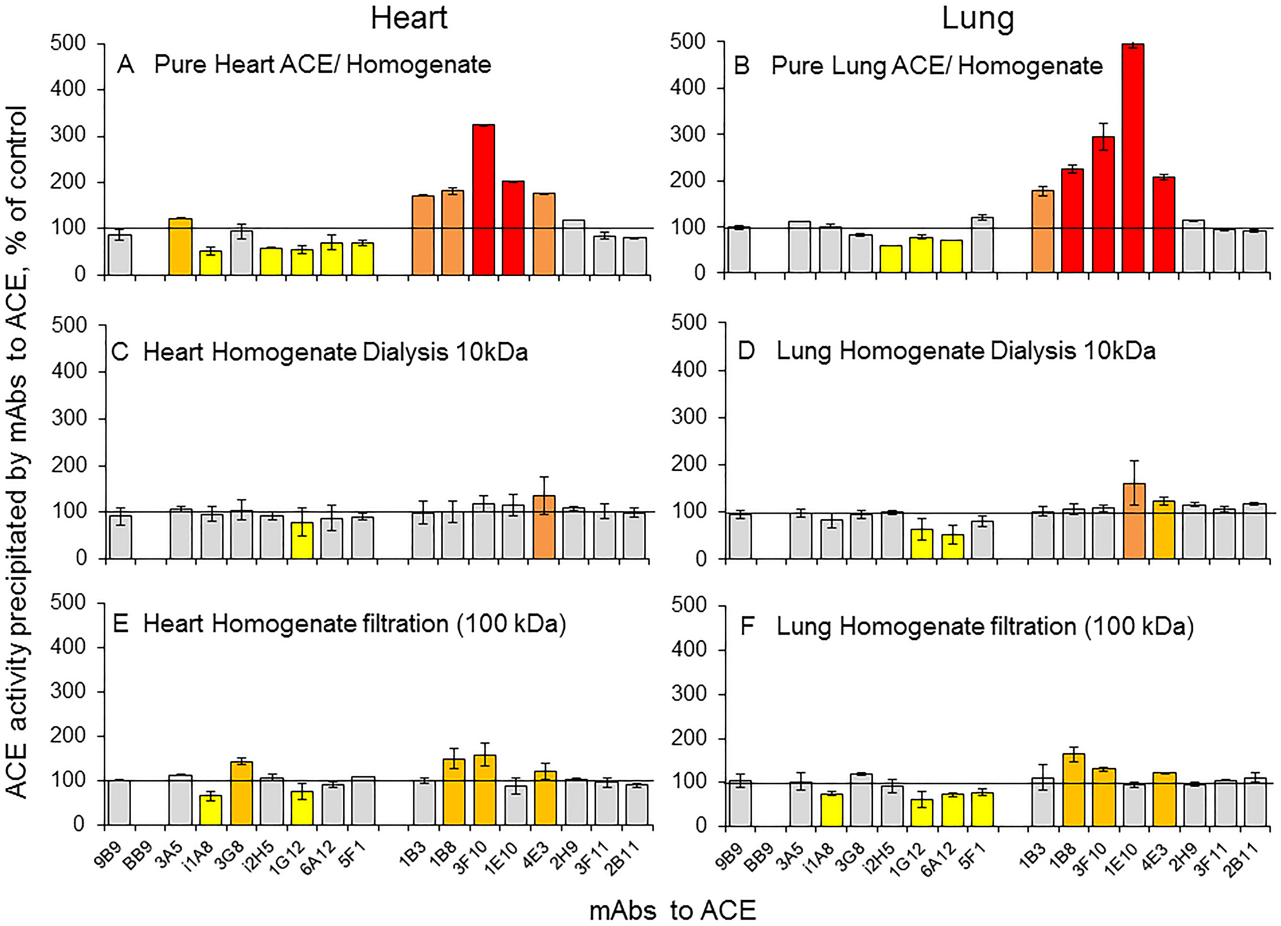
ACE effectors in the heart and lung tissues. Conformational fingerprinting of heart and lung ACEs was performed with a set of 17 mAbs to ACE as in the legend to Fig 3. Immunoprecipitated ACE activity after purification of heart and lung ACEs by anion-exchange and the affinity chromatography (**A,B**), after dialysis (**C, D**) and filtration through filter with 100 kDa limit (**E, F**) is presented as % (“binding ratio”) from immunoprecipitated ACE activity from the parent homogenates. Ratios increased more than 20% are highlighted in orange, more than 50% in dark orange, and more than 100% in red. Ratios decreased more than 20% are highlighted in yellow, more than 50% in deep blue. Data are mean ± SD of 2–3 experiments (each in duplicates), p<0.01.

As we discussed above, tissue homogenates contain high amounts of ACE inhibitors/effectors. When we performed purification of heart and lung ACEs by a combination of anion-exchange and affinity chromatography, we noticed significant influence of this purification procedure on the ACE conformational fingerprint (Fig 4A and 4B, S3A Fig, and “S2 Text”), that is a microenvironment significantly influenced on ACE conformation in the tested tissues. Purification of both ACEs from the corresponding homogenates led to a decrease of the binding of mAbs 1G12, 6A12, and i2H5 having overlapping epitopes on the N domain [35], which could be attributed to the dissociation of endogenous ACE inhibitors [26,35] from the complex with ACE.

In addition, several antibodies to the C domain, mAbs 1B3, 1B8, 3F10, 1E10, and 4E3, dramatically increased their binding to ACE as a result of the enzyme purification (Fig 4A and 4B). It is important that we did not observe such remarkable increase in the binding of these mAbs neither after dialysis (Fig 4C and 4D) nor after filtration of homogenates through filters with 30 kDa limit (not shown) or even 100 kDa limit (Fig 4E and 4F). These data allowed us to suggest that, in addition to the presence of endogenous easily-dissociating (due to dilution, dialysis or filtration) LMW ACE inhibitors (Figs 2 and 4C–4F) in both heart and lung tissues, these tissues contain some high molecular weight (HMW) ACE effectors/binding proteins, which could not be eliminated via filtration or dialysis but are eliminated during ACE purification procedures (“S3 Fig” and “S4 Fig”).

### Kinetic characteristics of heart and lung ACE

In order to determine whether these differences in conformation of heart and lung ACEs (Fig 3) are reflected in different functions of these ACEs, we compared kinetic characteristics of these enzyme.

**Fig 5:**
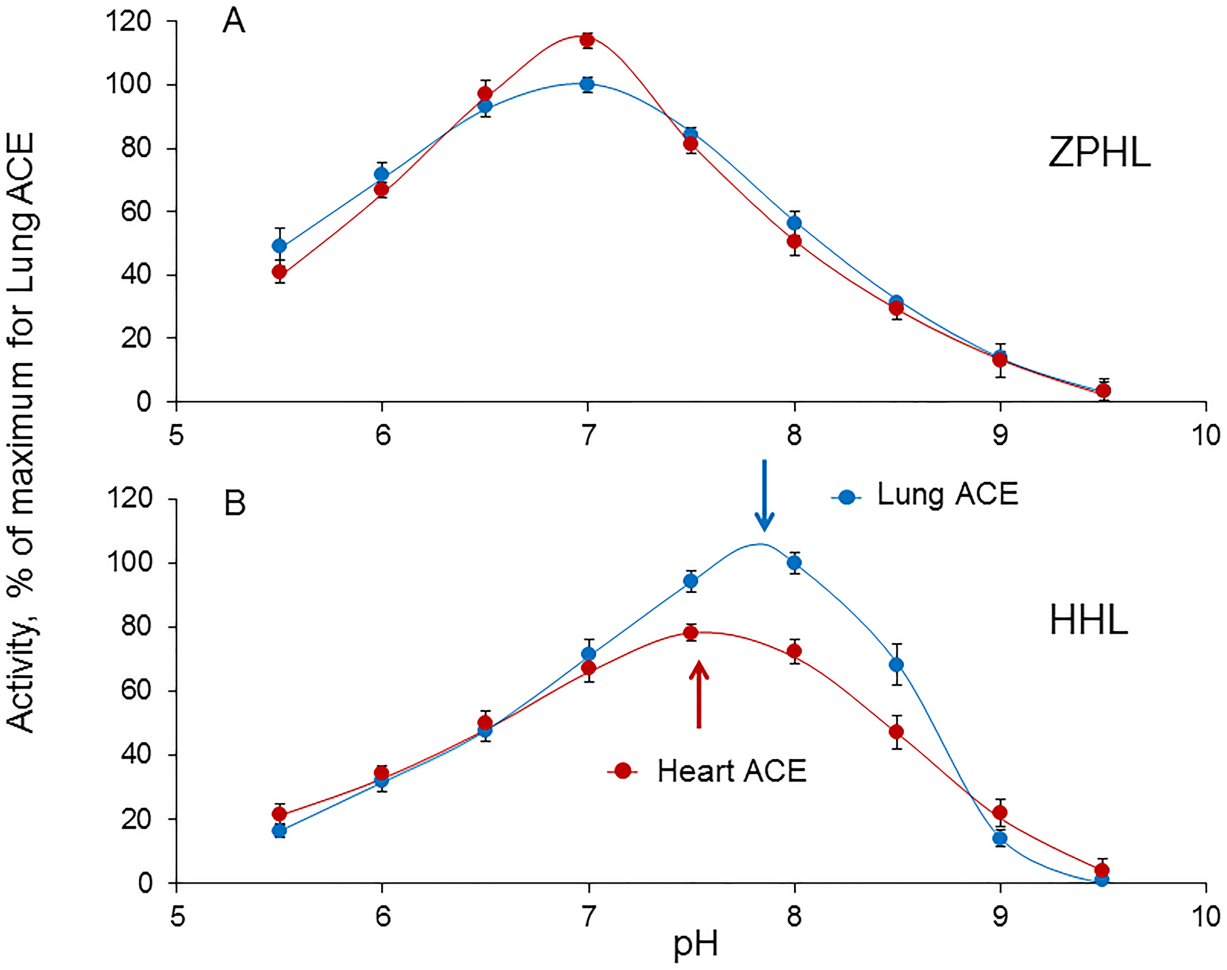
Dependence of ACE activity on pH. The assays of the activity of the purified heart and lung ACEs toward 0.5 mM Z-Phe-His-Leu (**A**) and 1.3 mM Hip-His-Leu (**B**) were performed in 25 mM acetate-MES-Tris-borate buffer, containing 0.15 M NaCl and 1 μM ZnCl_2_. Each value is a mean of several (2–3) experiments in duplicates.

The pH-dependencies of enzymatic activity of heart and lung ACEs in the hydrolysis of Z-Phe-His-Leu at 0.15 M NaCl were quite similar with pH-optimum about 7.0 (Fig 5) in accordance with previous results obtained for bovine ACE [18]. However, the pH-optima for the hydrolysis of Hip-His-Leu were equal to 7.5 for heart ACE and 7.8–7.9 for lung ACE (Fig 5), thus confirming different conformations of the two enzymes.

The inhibitory effect of anti-catalytic mAb 5F1 [21] to the N domain and mAbs 1E10 and 4E3 to the C domain [36], while these mAbs differently bound to the heart and lung ACEs (Fig 3A and 3B), did not show, however, significant difference in the extent of inhibition of these two ACEs (not shown).

The kinetic parameters, *k*_cat_ and K_m_, of the hydrolysis of several synthetic substrates, as well as a natural substrate angiotensin I, by purified heart and lung ACEs are presented in Table 1. Two of the substrates, Z-Phe-His-Leu and Hip-His-Leu, are C-terminal analogs of angiotensin I, while a substrate with Phe-Arg on its C-terminus could be considered as a C-terminal analog of another natural ACE substrate, bradykinin, as well as C-terminal analog of atriopeptin 2.

**Table 1:** Kinetic parameters of the hydrolysis of different substrates by ACEs isolated from human heart and lung tissues.

Substrate	Heart ACE			Lung ACE		
	k_cat_, s^-1^	K_m_, mM	k_cat_ /K_m_, s^-1^·mM^-1^	k_cat_, s^-1^	K_m_, mM	k_cat_ /K_m_, s^-1^·mM^-1^
Angiotensin I	22±1	0.04±0.01	535	21±3	0.04±0.01	518
Cbz-Phe-His-Leu	131±16	0.21±0.06	625	128±22	0.20±0.04	640
Hip-His-Leu	36±1	1.18±0.50	30	63±4	1.20±0.35	52
FA-Phe-Gly-Gly	220±18	0.24±0.07	980	173±8	0.24±0.04	730
FA-Phe-Phe-Arg	47±6	0.03±0.01	1657	46±2	0.02±0.01	1943

Assay conditions: 50 mM phosphate buffer, pH 7.5, containing 150 mM NaCl and 1 μM ZnCl_2_, 25°C. Error is represented as standard error of the mean (±SEM).

The absolute k_cat_ values calculated for the hydrolysis of angiotensin I by the human ACE markedly differ in the literature, from 3.5 s^-1^ [37] to 40 s^-1^ [38] for recombinant human ACE expressed in CHO cells, to 250 s^-1^ for human heart ACE [39], and to 66 s^-1^ for human kidney ACE (Kost OA, unpublished data), likely due to different conditions of angiotensin synthesis, different conditions of its hydrolysis, and different conditions and extent of ACEs purification. So, to minimize the putative errors, we determined the kinetic parameters of the hydrolysis of definite substrates by the two ACEs, from heart and lung, simultaneously.

The K_m_ values of the hydrolysis of any substrate by the two ACEs appeared to be similar, as well as the k_cat_ values for most substrates. However, for a short substrate Hip-His-Leu, the k_cat_ values of the hydrolysis differed two-fold for the heart and lung ACEs (Table 1).

Some differences in the catalytic properties of heart and lung ACEs were shown earlier for human [39] and bovine ACE [18], as well as different ability of these enzymes to be inhibited by a set of inhibitors [40]. There are some examples demonstrating the influence of glycosylation on the kinetic properties of the enzymes [41], and references herein, including ACE which activity could vary 2-fold due to different glycosylation [42,43]. Thus, it is likely that different ACE conformations induced by different protein glycosylation in different tissues could be the reason for different kinetic characteristics of these ACEs. We could not even exclude the possibility that unique ability to hydrolyze atriopeptin II which was reported only for heart, but not lung, ACE [39] could be partially explained by the differences in the conformations of the two ACEs due to different glycosylation.

### ACE phenotyping in different heart chambers

**Fig 6:**
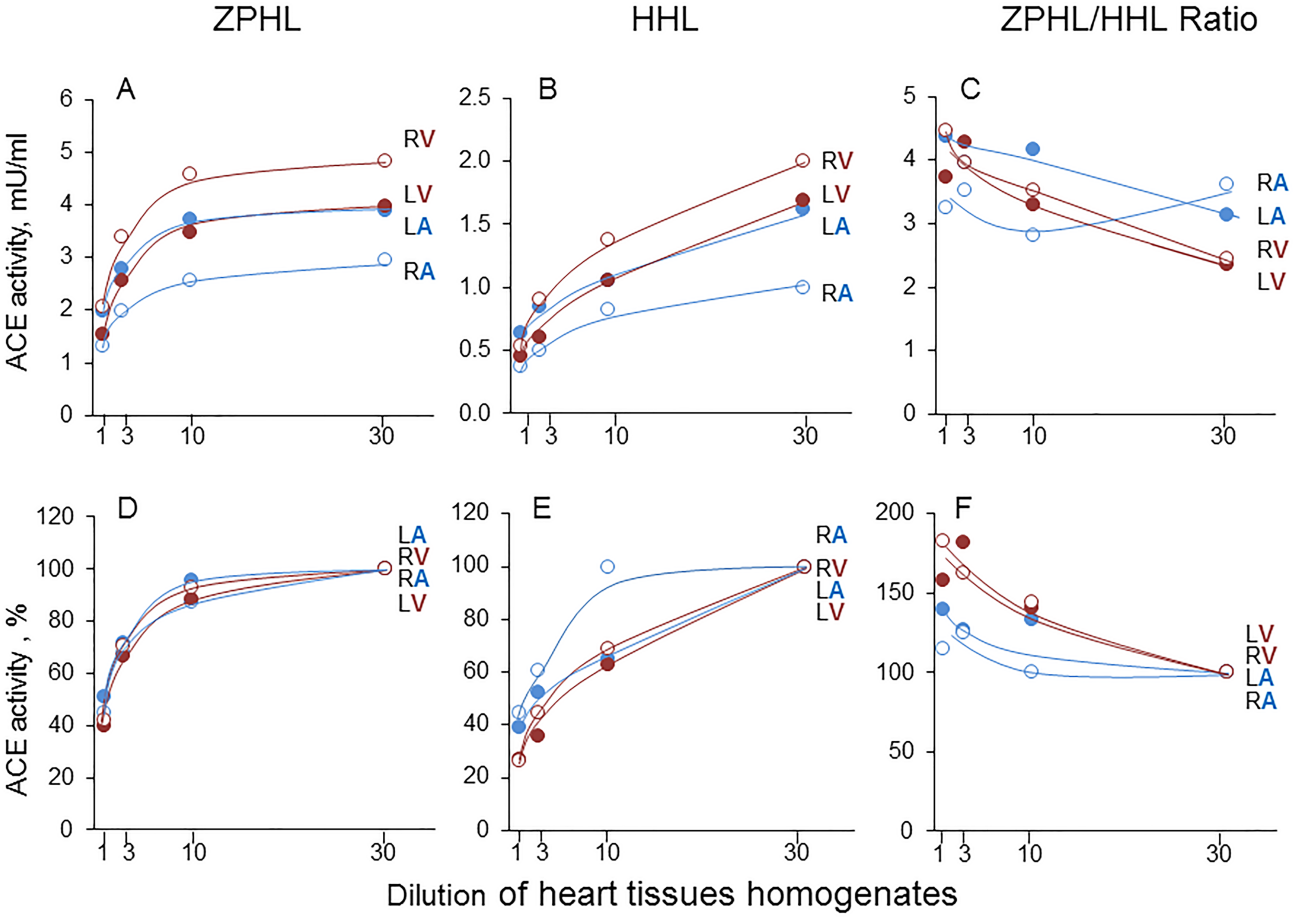
Effect of dilution on ACE activity in the homogenates of heart chambers. ACE activity and ZPHL/HHL ratio were measured in the homogenates of human heart chambers at different dilutions using two substrates (as in the legend to Fig 1). Data are expressed as absolute values (**A**-**C**) and as % from homogenates at maximal dilution—1/30 (**D**-**F**). Each value is a mean of several (2–3) experiments on separate homogenates in duplicates.

In order to estimate putative heterogeneity of ACE expression in heart chambers we performed ACE phenotyping in homogenates of the whole heart chambers from 10 donors. The presence of endogenous ACE inhibitors in heart chambers was demonstrated by the comparison of the apparent ACE activity in atria and ventricles at different dilutions (Fig 6). The apparent ACE activity in heart chambers markedly depended on homogenate dilution, as was observed for the whole heart and lung homogenates (Fig 2). Effect of homogenates dilution confirmed the lower apparent ACE activity in atria in comparison with ventricles (Fig 6A and 6B). The amount of inhibitors in heart chambers, however, varied in different donors. Bearing in mind that ventricles comprise the main part of the whole heart it is obvious that ventricle tissues are the main contributor to the amount of ACE inhibitors in the heart tissues (Fig 2).

**Fig 7:**
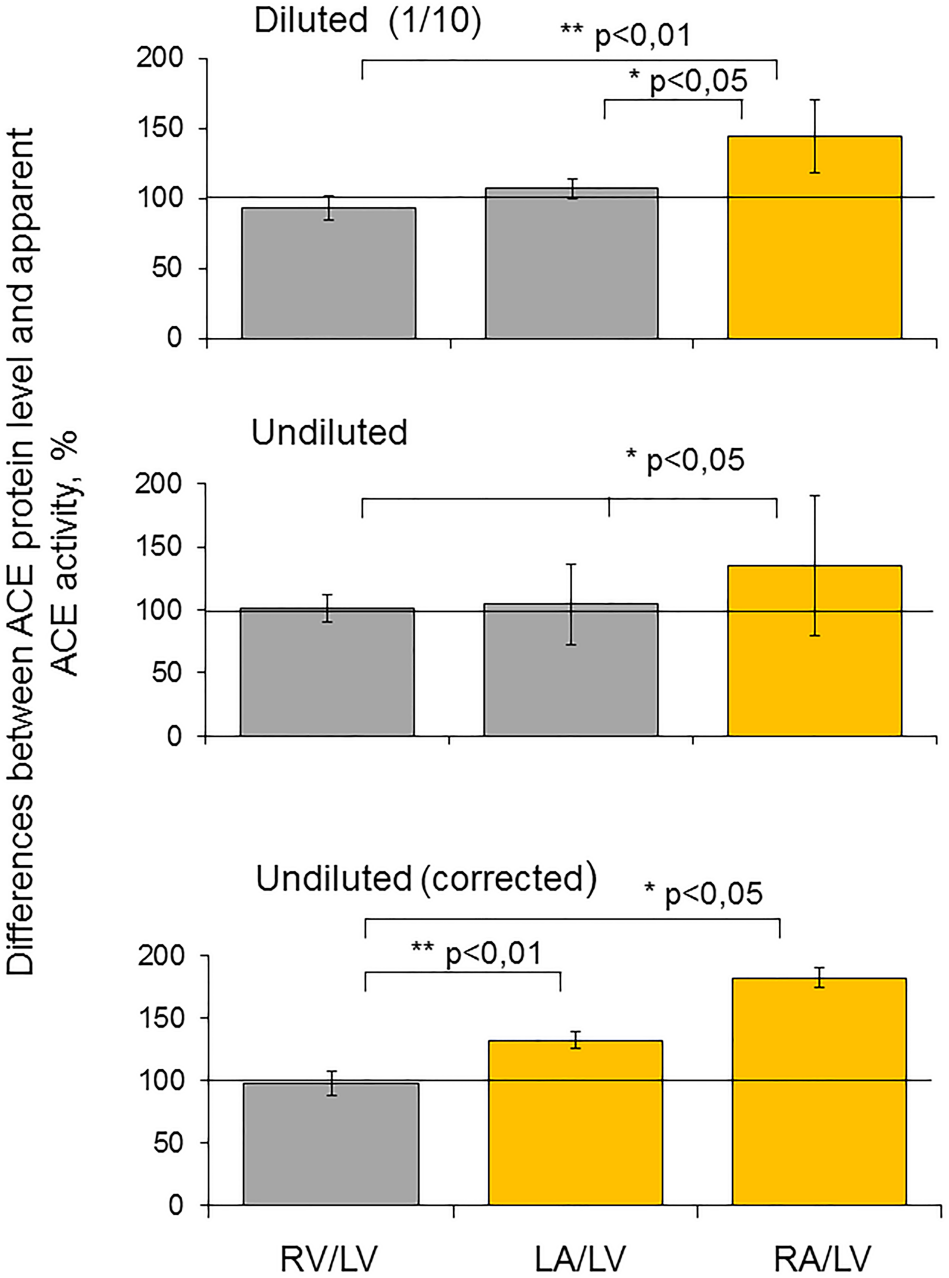
Differences between ACE protein level and ACE activity in the heart chambers. ACE activity was measured in the undiluted homogenates of human heart chambers and at 1/10 dilution as in Fig 1. ACE protein level was quantified after precipitation of ACE by a set of mAbs and washing out putative endogenous ACE inhibitors/effectors [26]. Data are expressed as a percentage from the corresponding data for left ventricle homogenate. Each value is a mean of several (3) experiments in duplicates. Bars highlighted in orange represent the samples with values higher than 20% of mean ± SD for control samples (left ventricle homogenate), * p<0.05, ** p<0.01.

When we compared the amount of immunoreactive ACE protein determined by precipitation of ACEs by mAb 9B9 [21] with an apparent ACE activity in different heart chambers, we have found that their ratio in the right atrium is significantly bigger than in other chambers (Fig 7), especially for undiluted homogenates, which is likely explained by the higher content of ACE inhibitors in the atrium.

**Fig 8:**
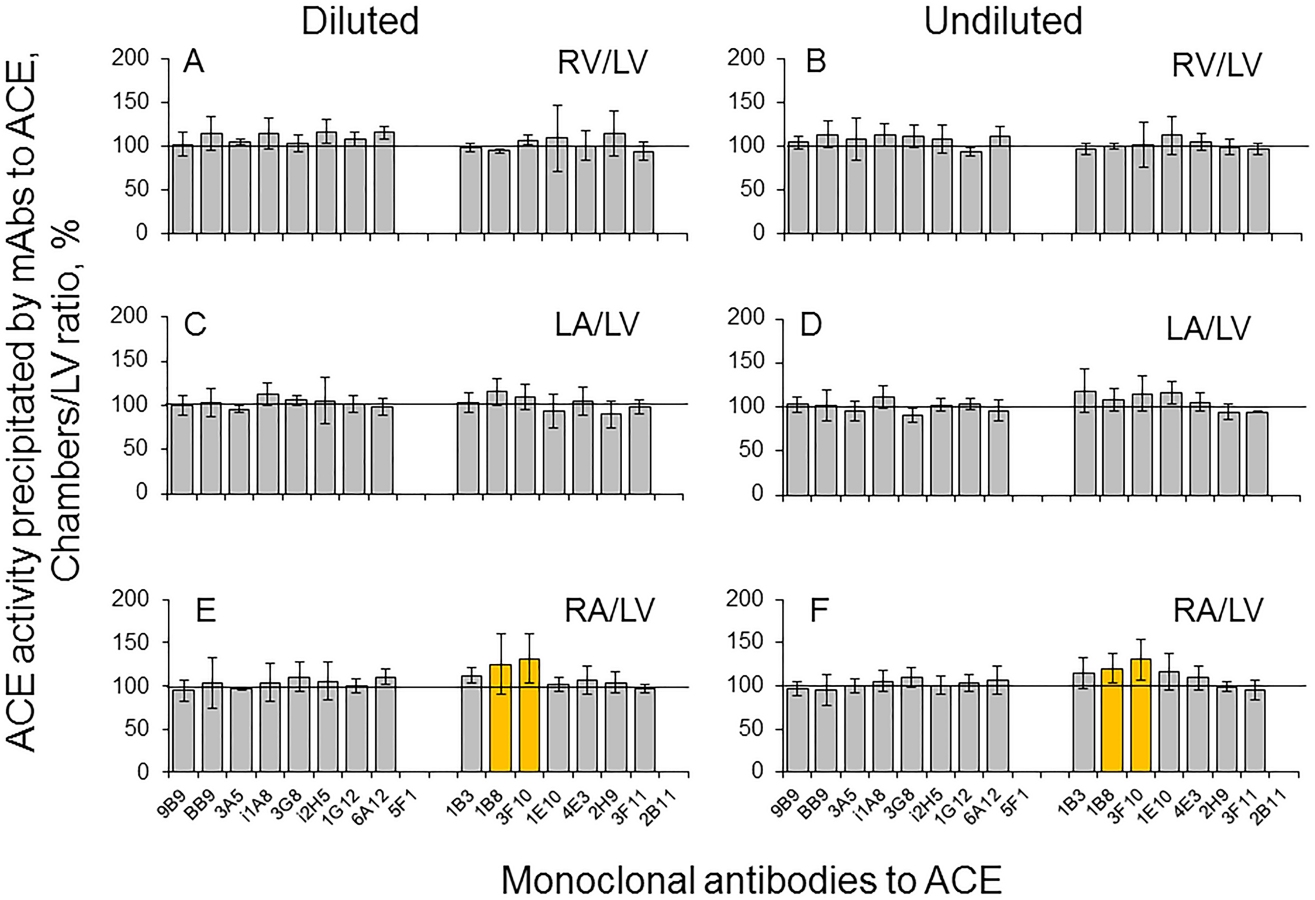
Conformational fingerprinting of ACE in different heart chambers. Conformational fingerprinting of ACE in the homogenates (1:9) of different heart chambers was performed with a set of 17 mAbs to ACE as in Fig 3. Immunoprecipitated ACE activity from these homogenates undiluted and further diluted 1/10 is presented as % (“binding ratio”) from the immunoprecipitated ACE activity from left ventricle homogenate (undiluted and diluted, correspondingly). Ratios increased more than 20% are highlighted in orange. Data are mean ± SD of at least 3 experiments (each in duplicates), p<0.01.

Conformational fingerprinting of ACE in heart chambers (Fig 8) revealed statistically significant increased binding of mAbs 1B8 and 3F10 to ACE from the right atrium (Fig 8E and 8F), while the difference in the binding of mAbs to ACE from the right ventricle or the left atrium (Fig 8A–8D) was negligible.

This difference was observed both in undiluted and diluted homogenates, which means that the reason is the conformational difference between ACEs expressed in atrium and ventricle, not the presence of different effectors. Moreover, conformational fingerprinting of ACE was separately performed on 10 sets of homogenates of heart chambers obtained from 10 donors for reproducibility and statistics. These two mAbs, 1B8 and 3F10, have highly overlapping epitopes on the C domain which contain potential glycosylation site Asn731 [17,36]. Highly probable that different microenvironment of ACE in the heart chambers (Figs 6 and 7) can lead to different glycosylation of Asn731 in ACE in the right atrium, compared to the glycosylation of this particular glycosylation site in ACE in other chambers, and, as a results of that, lead to different effectiveness of the binding of mAbs 1B8 and 3F10.

It is worth noting that atrial tissues are likely to perform specific biochemical functions. As an example, only atrial bovine tissue was reported to contain a metallodipeptidyl carboxyhydrolase (EC 3. 4.15.4) which resembles ACE, as it is able to hydrolyze ACE common substrate Hip-His-Leu and be inhibited by ACE inhibitors. This enzyme, however, differed from ACE in a number of molecular and kinetic properties, the major difference being the ability to hydrolyze atriopeptin II, which could be considered as heart-specific substrate, and its analogue Hip-Ser-Phe-Arg [44], and references herein.

Thus, there could be major differences in the biochemical processes within the heart chambers due to different enzymatic content. However, there could be also more subtle differences due to the changed properties of the same enzyme expressed in different heart chambers.

### Conclusions

The significant differences in the local conformations of heart ACE (originated from heart endothelial cells and likely from myofibroblasts) and lung ACE (originated from lung endothelial cells) allow us to suggest that the properties and functions of ACE could be sensitive to the microenvironment and be regulated by accompanying constituents of tissues and blood, this regulation could depend on a set of possible ACE effectors in heart and lung tissues.

Therefore, significant structural differences in ACE from heart and ACE from lung demonstrated in this study may be the base for the generation of mAbs, which will distinguish these two ACEs. Possible limitation of this study is that we did not generated yet these mAbs. We have to say that this is a pioneer and extremely difficult work because the discriminative power of such mAbs should be very high (about 100-fold) in order to detect only about 1% of heart-derived ACE in the blood where the majority of ACE molecules comes from lung. Therefore, the occurrence of hybridomas with required specificity would be low demanding to test thousands of hybridomas producing anti-ACE mAbs.

**Fig 9:**
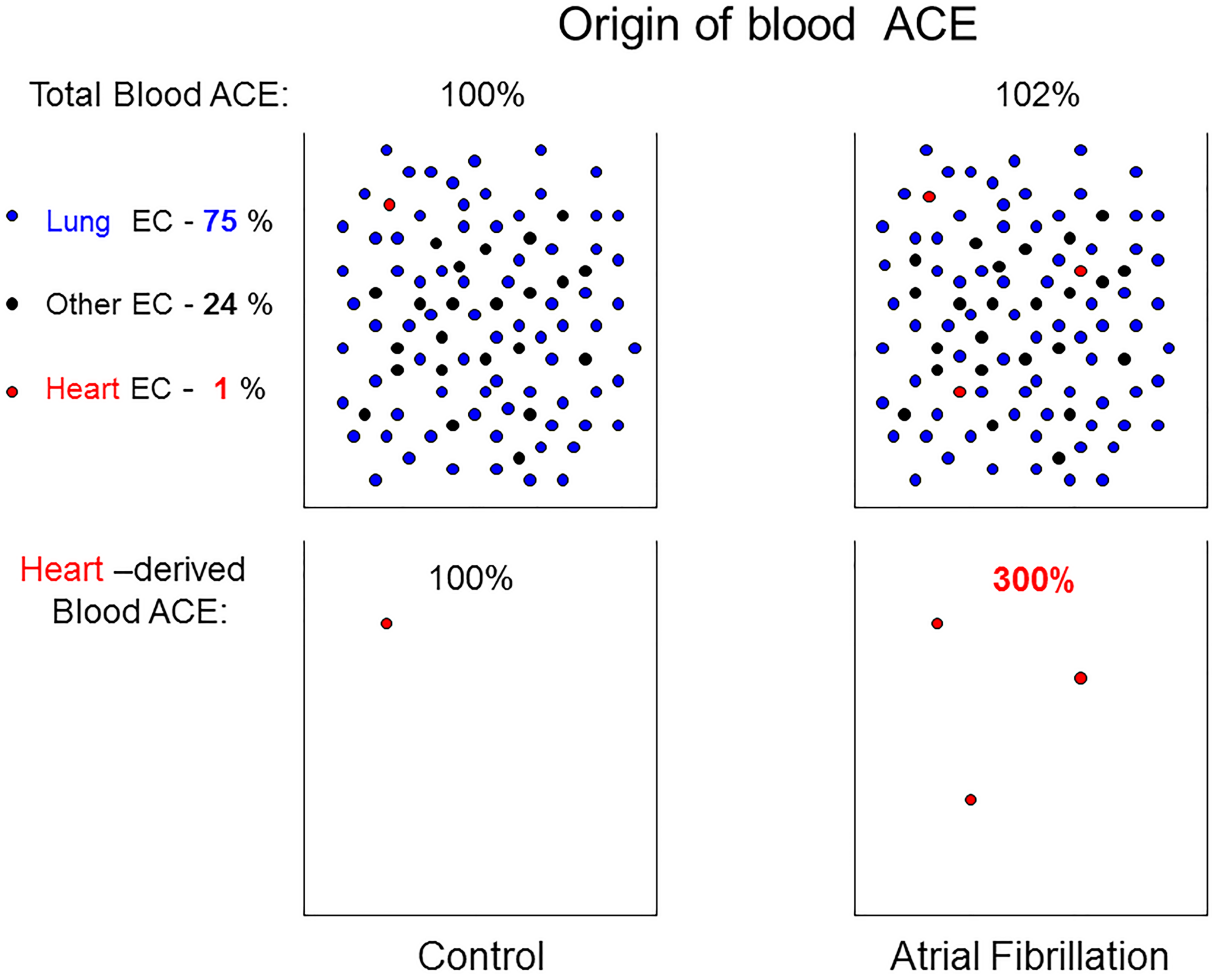
Hypothetical scheme of the assay for quantification of heart-derived ACE in the blood. According to our estimation (based on heterogeneous ACE expression in capillary endothelial cells of different organs [15]) lung ACE provides about 75% of blood ACE, whereas heart-derived ACE activity in the blood could not be more than 1%. Therefore, overall increase in blood ACE as a result of 3-fold increase in the heart ACE due to atrial fibrillation [2] will not increase substantially blood ACE activity in these patients. However, precipitation and quantification from the blood of heart-derived ACE by mAbs, specific for heart ACE, may be diagnostically relevant.

However, such mAbs (if we will be able to select them) may have a potential for the development of blood-based assay for quantification of heart-derived ACE in the blood for identification of the patients with the increased level of heart ACE, i.e. with the increased risk of atrial fibrillation, which cannot be achieved by estimation of ACE activity in the blood (Fig 9).

## Supporting information

S1 Table Purification of ACE from human heart tissue. (DOCX)

S1 Fig SDS-PAGE electrophoresis of heart and lung ACE. Samples of ACEs were prepared and analysed in 7.5% SDS-PAGE. Lane 1, purified heart ACE (3.8 μg); lane 2, gel electrophoresis molecular weight markers; lane 3, purified lung ACE (7.2 μg). Lanes were stained with Coomassie Brilliant Blue.
(TIF)

S2 Fig Effect of ACE perfusion via rat circulation. We equilibrated seminal fluid ACE (**A**) and purified heart (**B**) and lung (**C**) ACEs by activity and injected 1000 mU of each enzyme in 100 μl into rat tail veins. After 30 min circulation, the blood was collected and citrated plasma prepared. Conformational fingerprinting of human ACE was performed as in a legend to Fig 3. Immunoprecipitated ACE activity after perfusion into rat blood circulation is presented as % (“binding ratio”) from that for purified ACE. Ratios increased more than 20% are highlighted in orange, and more than 50% in dark orange. Ratios decreased more than 20% are highlighted in yellow, more than 50% in deep blue.
(TIF)

S3 Fig Effect of heart homogenate components on mAbs binding to human heart ACE. Conformational fingerprinting of the heart ACE was performed with a set of 17 mAbs to the two-domain ACE as in the legend to Fig 3. **A**. The influence of ACE purification by anion-exchange and affinity chromatography on mAbs binding. **B** and **C**. The effect of fractionated heat-inactivated (65°, 30 min) flow-through obtained at anion-exchange chromatography of ACE homogenate on the binding of mAbs with purified ACE, represented as a percentage from that for purified ACE. **D**. The effect of specific ACE inhibitor enalaprilat on mAbs binding to the heart ACE.
(TIF)

S4 Fig The putative area on the C domain of ACE for putative HMW(-s) effectors. The epitopes for mAbs 1B3, 1B8, 3F10, 1E10 and 4E3 were mapped on the surface of the C domain of human ACE (PDB 1OC2) according to [36]. The positions of the N- and C-ends of the C domain and some amino residues on the surface of the enzyme are shown for orientation. Potential glycosylation sites are marked by green. While mAbs 1B3, 1B8, 3F10 have overlapping epitopes near the C-end of the C domain, another pair of mAbs, 1E10 and 4E3, have their epitopes near its N-end. Thus, we suggested that HMW effector(-s) forms complexes with ACE covering the region on the surface of the C domain of ACE between the epitopes for all these mAbs. The putative area of the contact of ACE with HMW effector from tissue homogenates is presented as a red ellipse.
(TIF)

S1 Text Apparent aminopeptidase activity in the heart. (DOCX)

S2 Text Effect of heart homogenate components on mAbs binding to human heart ACE. (DOCX)

## Abbreviations

ACE: angiotensin-converting enzyme
AF: atrial fibrillation
FA-: furanacryloyl-
HHL (Hip-His-Leu): hippuryl-L-histidyl-L-leucine
HMW: high molecular weight
LMW: low molecular weight
PTM: post translational modifications
RAS: renin-angiotensin system
ZPHL (Z-Phe-His-Leu): carbobenzoxy-L-phenylalanyl-L-histidyl-L-leucine
