# Supplementary material for: ACE phenotyping in human heart
Source: PLoS One. 2017 Aug 3;12(8):e0181976. doi: 10.1371/journal.pone.0181976 (PMC5542439; doi:10.1371/journal.pone.0181976)
Supplement: S1 Table — (DOCX) [file pone.0181976.s001.docx]

**S1 Table.  Purification of ACE from human heart tissue.**

| Stage | Protein, mg | Activity, mU | Sp.activity, mU/mg | Purification factor | Yield, % |
| --- | --- | --- | --- | --- | --- |
| 1. Extraction | 8525 | 4489 | 0,53 | 1 | 100 |
| 2. Anion-exchange chromatography | 1674 | 4080 | 2,44 | 4,6 | 91 |
| 3. Affinity chromatography | 0,332 | 2332 | 7024 | 13339 | 52 |
| 4. Ultrafiltration 100kDa | 0,035 | 1262 | 36049 | 68459 | 28 |

*ACE was purified from 274 g of human heart tissue as described in Experimental section. Activity was assayed with Z-Phe-His-Leu (2 mM) as substrate.

ACE purification from lung homogenates was performed by anion-exchange chromatography on DEAE-Toyopearl 650M and then lisinopril affinity chromatography [18]. As a result, we obtained electrophoretically purified lung ACE. Of note, previous purifications of ACE from bovine lung did not included anion-exchange chromatography but included protein precipitation by ammonium sulfate [45]. However, this approach appeared to be non-suitable for human ACE because of substantial loss of ACE specific activity during such treatment. Thus, we choose anion-exchange chromatography as a preliminary purification procedure before affinity chromatography.

However, ACE purification from heart homogenates by the same protocol, anion-exchange chromatography and lisinopril affinity chromatography, appeared to be less efficient likely due to the substantially lower level of ACE in heart tissues (Fig 1). Electrophoretically pure heart ACE (S1 Fig, lane 1) was obtained only after additional 4 cycles of concentration-dilution (“washing”) of ACE on ultrafiltration membranes with 100 kDa pores.

Reference

1. [Kost OA](https://www.ncbi.nlm.nih.gov/pubmed/?term=Kost%20OA%5BAuthor%5D&cauthor=true&cauthor_uid=11114069), [Bovin NV](https://www.ncbi.nlm.nih.gov/pubmed/?term=Bovin%20NV%5BAuthor%5D&cauthor=true&cauthor_uid=11114069), [Chemodanova EE](https://www.ncbi.nlm.nih.gov/pubmed/?term=Chemodanova%20EE%5BAuthor%5D&cauthor=true&cauthor_uid=11114069), [Nasonov VV](https://www.ncbi.nlm.nih.gov/pubmed/?term=Nasonov%20VV%5BAuthor%5D&cauthor=true&cauthor_uid=11114069), [Orth TA](https://www.ncbi.nlm.nih.gov/pubmed/?term=Orth%20TA%5BAuthor%5D&cauthor=true&cauthor_uid=11114069). New feature of angiotensin-converting enzyme: carbohydrate-recognizing domain. J Mol Recognit. 2000;13: 360-369.
