# Supplementary figures and images for: ACE phenotyping in human heart
Source: PLoS One. 2017 Aug 3;12(8):e0181976. doi: 10.1371/journal.pone.0181976 (PMC5542439; doi:10.1371/journal.pone.0181976)

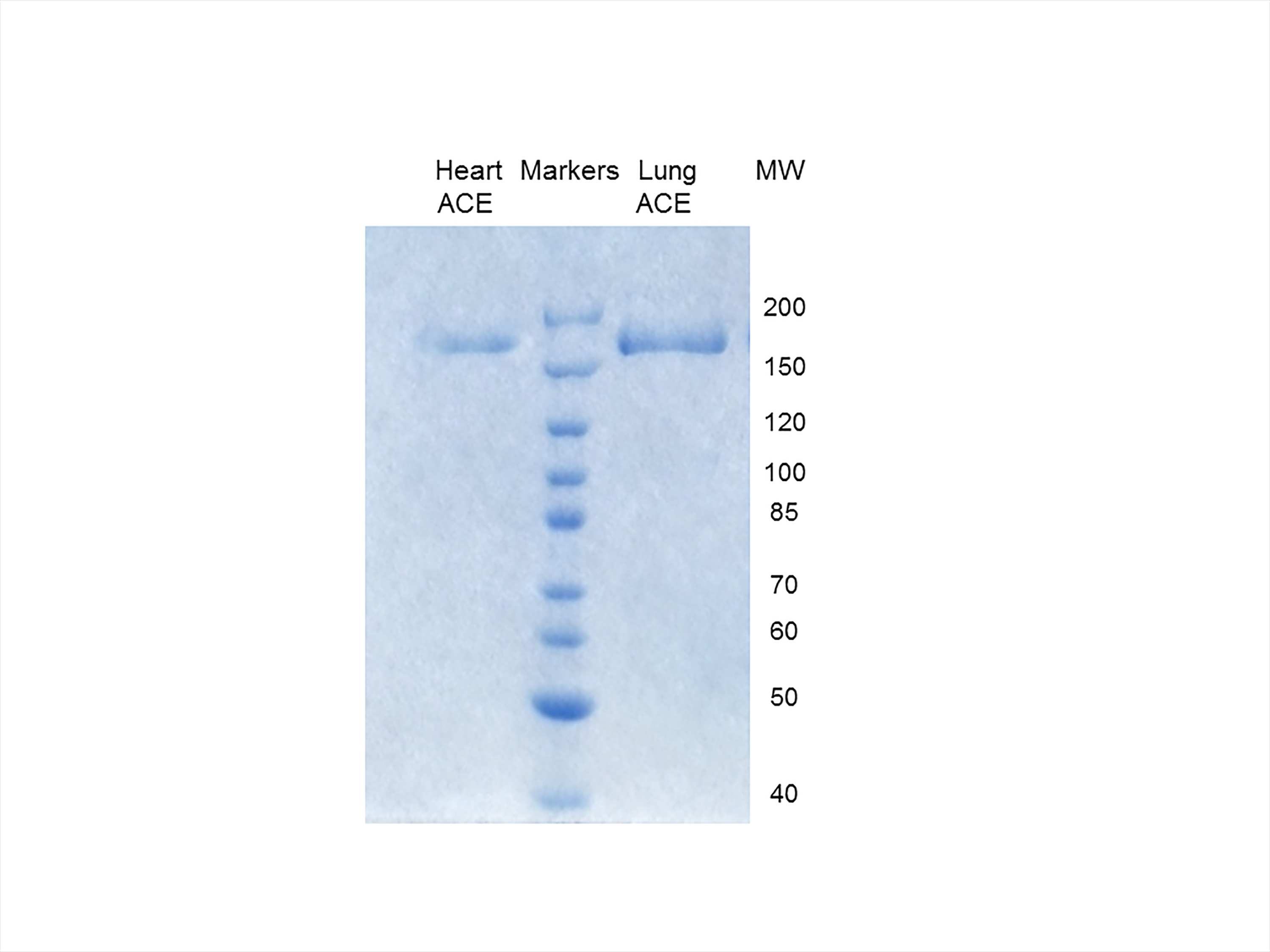

Supplement: S1 Fig — Samples of ACEs were prepared and analysed in 7.5% SDS-PAGE. Lane 1, purified heart ACE (3.8 μg); lane 2, gel electrophoresis molecular weight markers; lane 3, purified lung ACE (7.2 μg). Lanes were stained with Coomassie Brilliant Blue. (TIF) [file pone.0181976.s002.tif]

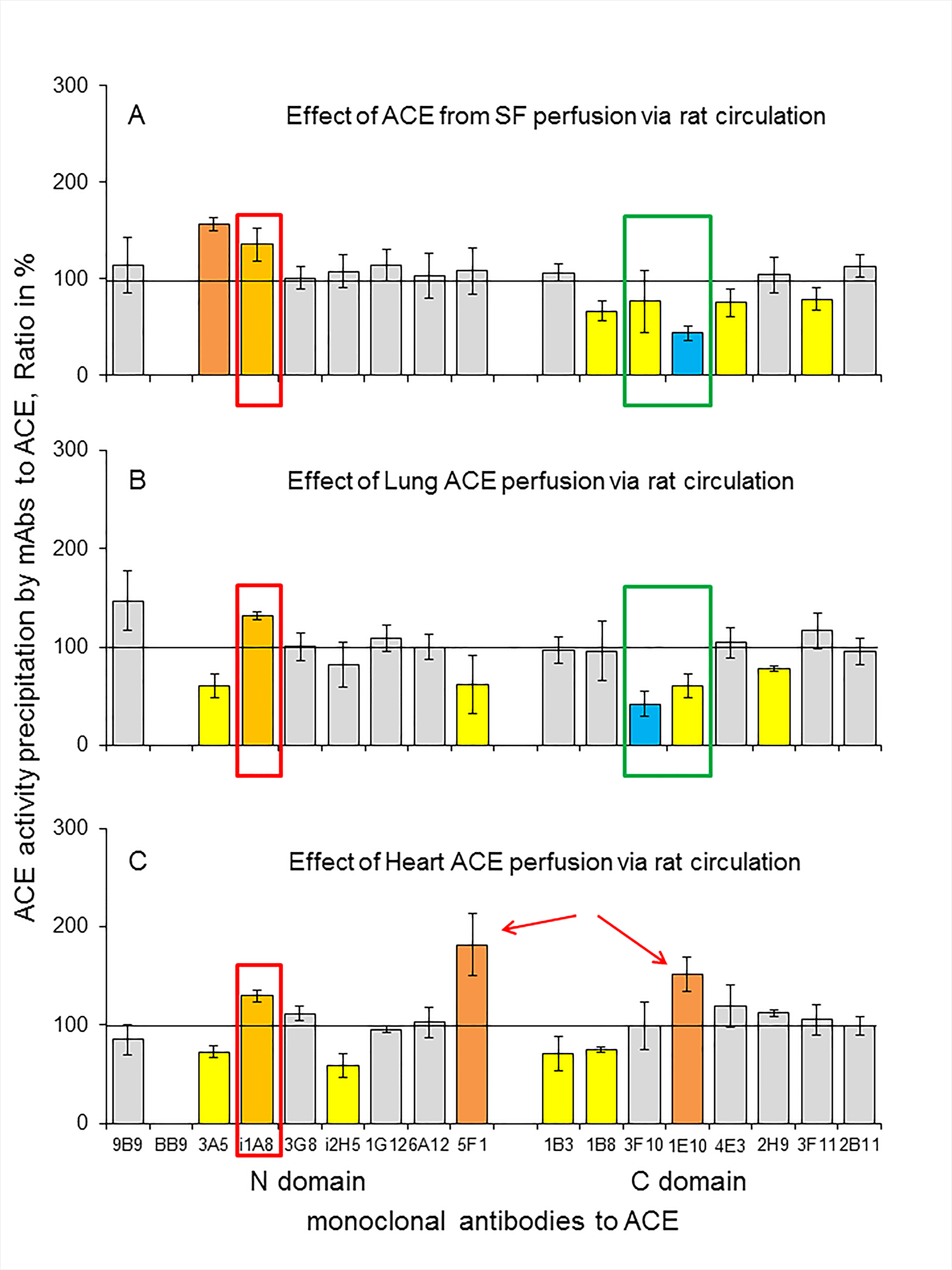

Supplement: S2 Fig — We equilibrated seminal fluid ACE (A) and purified heart (B) and lung (C) ACEs by activity and injected 1000 mU of each enzyme in 100 μl into rat tail veins. After 30 min circulation, the blood was collected and citrated plasma prepared. Conformational fingerprinting of human ACE was performed as in a legend to Fig 3. Immunoprecipitated ACE activity after perfusion into rat blood circulation is presented as % (“binding ratio”) from that for purified ACE. Ratios increased more than 20% are highlighted in orange, and more than 50% in dark orange. Ratios decreased more than 20% are highlighted in yellow, more than 50% in deep blue. (TIF) [file pone.0181976.s003.tif]

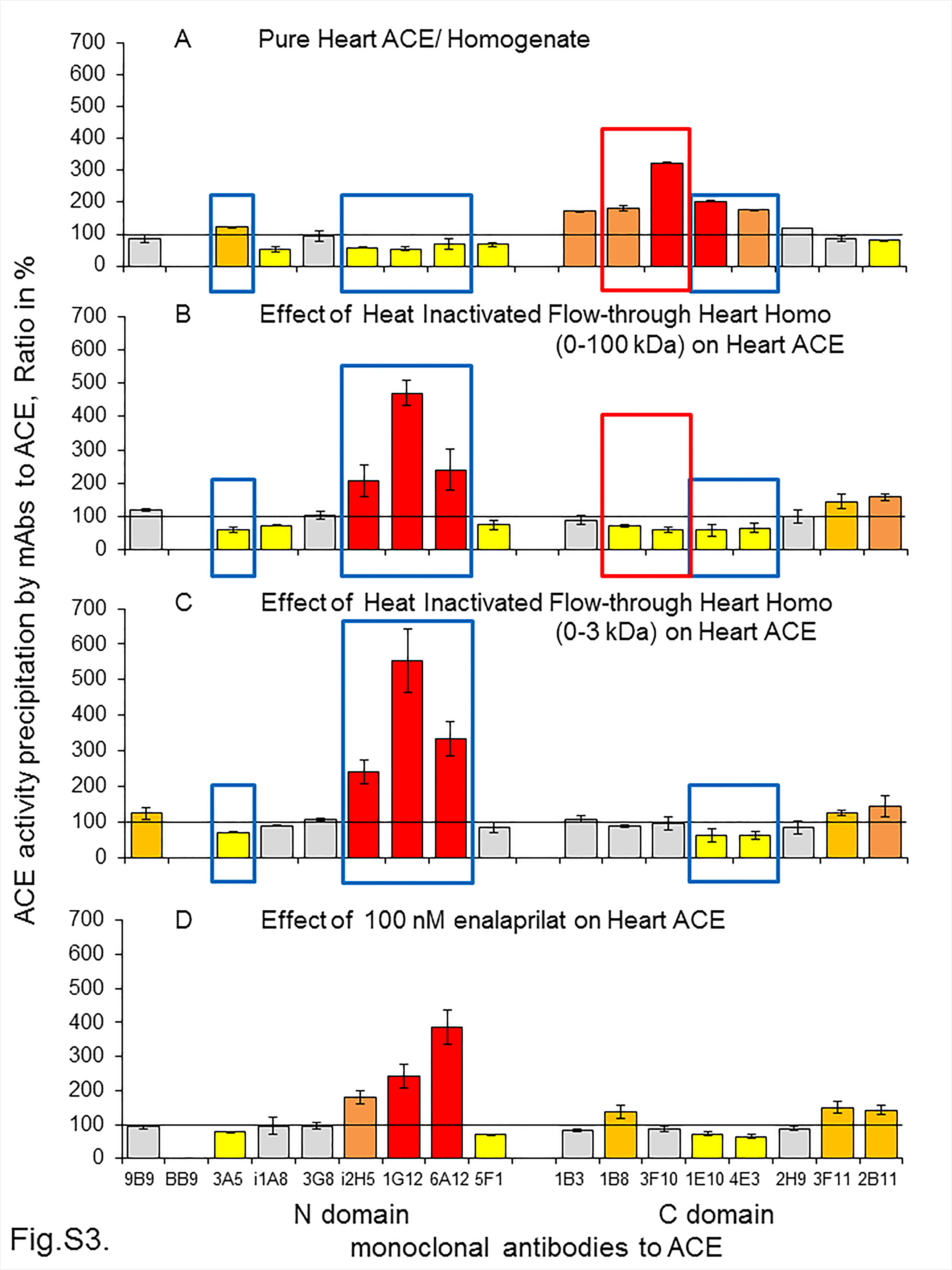

Supplement: S3 Fig — Conformational fingerprinting of the heart ACE was performed with a set of 17 mAbs to the two-domain ACE as in the legend to Fig 3. A. The influence of ACE purification by anion-exchange and affinity chromatography on mAbs binding. B and C. The effect of fractionated heat-inactivated (65°, 30 min) flow-through obtained at anion-exchange chromatography of ACE homogenate on the binding of mAbs with purified ACE, represented as a percentage from that for purified ACE. D. The effect of specific ACE inhibitor enalaprilat on mAbs binding to the heart ACE. (TIF) [file pone.0181976.s004.tif]

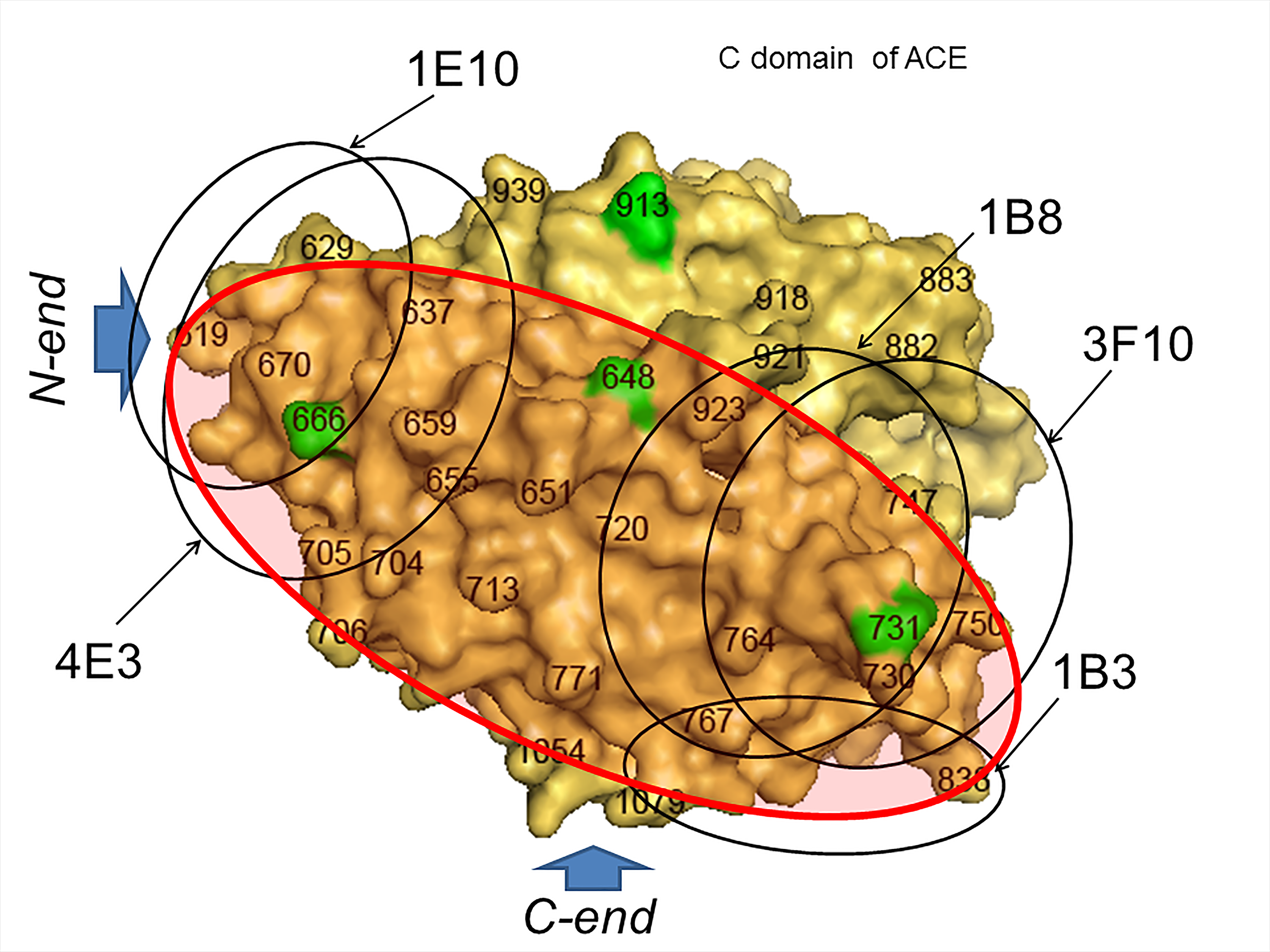

Supplement: S4 Fig — The epitopes for mAbs 1B3, 1B8, 3F10, 1E10 and 4E3 were mapped on the surface of the C domain of human ACE (PDB 1OC2) according to [36]. The positions of the N- and C-ends of the C domain and some amino residues on the surface of the enzyme are shown for orientation. Potential glycosylation sites are marked by green. While mAbs 1B3, 1B8, 3F10 have overlapping epitopes near the C-end of the C domain, another pair of mAbs, 1E10 and 4E3, have their epitopes near its N-end. Thus, we suggested that HMW effector(-s) forms complexes with ACE covering the region on the surface of the C domain of ACE between the epitopes for all these mAbs. The putative area of the contact of ACE with HMW effector from tissue homogenates is presented as a red ellipse. (TIF) [file pone.0181976.s005.tif]
