# Supplementary material for: ACE phenotyping in human heart
Source: PLoS One. 2017 Aug 3;12(8):e0181976. doi: 10.1371/journal.pone.0181976 (PMC5542439; doi:10.1371/journal.pone.0181976)
Supplement: S1 Text — (DOCX) [file pone.0181976.s006.docx]

**S1 Text. Apparent Aminopeptidase activity in the heart.**

We measured ACE activity in tissue homogenates in the presence of specific ACE inhibitor enalaprilat and showed that the substrates, Z-Phe-His-Leu and Hip-His-Leu, were not cleaved by any other peptidase with generation of dipeptide His-Leu. An apparent ACE activity depended on the dilution of tissue homogenates (Fig 2). One of the possible reasons for the effect of the decreased ACE activity in tissue homogenates could be the presence of aminopeptidase in human tissues able to hydrolyze the product of ACE enzymatic reaction, His-Leu, and, thus, decrease its concentration. Indeed, we showed that both heart and lung homogenates contained rather high amounts of aminopeptidase, which hydrolyzed dipeptide His-Leu added to tissue homogenates by purpose. For instance, incubation of 20 ul of undiluted heart homogenate with 100 ul of 0.01 mM His-Leu for 40 min led to the 50% hydrolysis of the peptide. The activity of this aminopeptidase (hydrolysis of His-Leu) was inhibited by inhibitor bestatin.

When we measured ACE activity in homogenates, however, the addition of bestatin was not needed as the presence of bestatin in the reaction media did not influence on the detected ACE activity. We carried out an additional experiment, in which ACE activity in homogenates was preliminarily inhibited by enalaprilat, and showed that His-Leu added to homogenate was not hydrolyzed by aminopeptidase in the presence of high concentrations of ACE substrates, 2 mM Z-Phe-His-Leu and 5 mM Hip-His-Leu. So, these peptides could be considered as inhibitors of aminopeptidase and we could disregard the cleavage of His-Leu by aminopeptidase in tissue homogenates in experimental conditions. Therefore, the presence of aminopeptidase is not a reason for the decrease of an apparent ACE activity in undiluted homogenates of human tissues, which should be attributed to the presence of endogenous ACE inhibitors in heart and lung tissues.
