# Supplementary material for: ACE phenotyping in human heart
Source: PLoS One. 2017 Aug 3;12(8):e0181976. doi: 10.1371/journal.pone.0181976 (PMC5542439; doi:10.1371/journal.pone.0181976)
Supplement: S2 Text — (DOCX) [file pone.0181976.s007.docx]

**S2 Text. Effects of heart homogenate components on ACE conformation.**

We determined that not only affinity chromatography but also single anion-exchange chromatography of ACE homogenates resulted in the remarkable increase of the binding of mAbs 1B3, 1B8, 3F10, 1E10, and 4E3 to the C domain. As both heart and lung ACEs were solubilized in the presence of Triton X-100 which is mainly eliminated during chromatography, we checked possible interfering effect of Triton on the binding of mAbs to ACE due to interaction of detergent with some part of ACE molecules maintaining the hydrophobic anchor. We found that the binding of mAbs 1E10 and 4E3 to purified ACE slightly decreased in the presence of Triton (p<0.05), but the binding of mAbs 1B3, 1B8 and 3F10 was unchanged (not shown). Thus, the difference in the binding of mAbs 1B3, 1B8, 3F10, 1E10, and 4E3 should not be referred to the presence of Triton X-100 in homogenates but to the presence of some ACE effector(-s) instead.

The flow-through obtained at anion-exchange chromatography of heart homogenate was heated at 65^°^ for 30 min for inactivation of unbound ACE and filtrated through 3 kDa and 100 kDa filters in order to obtain fractions containing LMW or LMW plus HMW components. The action of these fractions on the effectiveness of mAbs binding to purified heart ACE is presented on “S3B,C Fig” Filtrate containing LMW compounds significantly influenced mAbs binding to the N domain of ACE (“S3C Fig”) similar to the effect of a common ACE inhibitor, enalaprilat “S3D Fig” shown by blue boxes in “S3 Fig”.

It is worth noting that the effect of the inhibitor on mAbs 1G12/6A12 binding to purified heart ACE, about 400 %, is comparable to that obtained for ACE in human plasma [26,34,44], while the effect of enalaprilat on mAbs binding to lung, seminal fluid or recombinant ACEs was more modest, about 50 % [26,35]. The significant effect of enalaprilat on mAbs binding to plasma ACE is mainly explained by the dissociation of ACE effectors (lysozyme and bilirubin) present in plasma from the complex with ACE [26], which is induced by ACE conformational changes upon inhibitor binding. Lung and seminal fluid are likely lack of these effectors, while recombinant ACE does not have these effectors by definition. The fact that the effect of enalaprilat on mAbs binding to heart ACE is so huge implies more pronounced conformational changes in heart ACE molecule compared to lung ACE, which once again indicates on the differences in these two enzymes.

Filtrate containing both LMW and HMW components, in addition, caused significant decrease of the binding of mAbs 1B8 and 3F10 to the purified ACE “S3 Fig “ which should be attributed to the action of HMW effector(-s) in this filtrate, shown by red boxes.
